# Supplementary material for: ABA Enhances Drought Resistance During Rapeseed (Brassica napus L.) Seed Germination Through the Gene Regulatory Network Mediated by ABA Insensitive 5
Source: Plants (Basel). 2025 Apr 22;14(9):1276. doi: 10.3390/plants14091276 (PMC12073310; doi:10.3390/plants14091276)
Supplement: Supplementary file 1 [file plants-14-01276-s001.zip › Supplementary Figure S2.pdf]

BnA05g0194800.1-1 TTTGGTCTGAGATAC-TAG**AGG**

WT-1 TTTGGTCTGAGATACATAG**AGG**

BnA05g0194800.1-2 GTATGGAGTTGATATGG-A**GGG**

WT-2 GTATGGAGTTGATATGGGA**GGG**

BnC04g0676540.1-1 TTTGGTCTGAGATAC-TAG**AGG**

WT-1 TTTGGTCTGAGATACATAG**AGG**

BnC04g0676540.1-2 GTATGGAGTTGATATGG-A**GGG**

WT-2 GTATGGAGTTGATATGGGA**GGG**

BnA04g0179250.1-1 TTTGGTCTGAGATAC-TAG**AGG**

WT-1 TTTGGTCTGAGATACATAG**AGG**

BnA04g0179250.1-2 GTATGGAGTTGATATGG-A**GGG**

WT-2 GTATGGAGTTGATATGG**GA****GGG**
