# Supplementary figures and images for: ABA Enhances Drought Resistance During Rapeseed (Brassica napus L.) Seed Germination Through the Gene Regulatory Network Mediated by ABA Insensitive 5
Source: Plants (Basel). 2025 Apr 22;14(9):1276. doi: 10.3390/plants14091276 (PMC12073310; doi:10.3390/plants14091276)

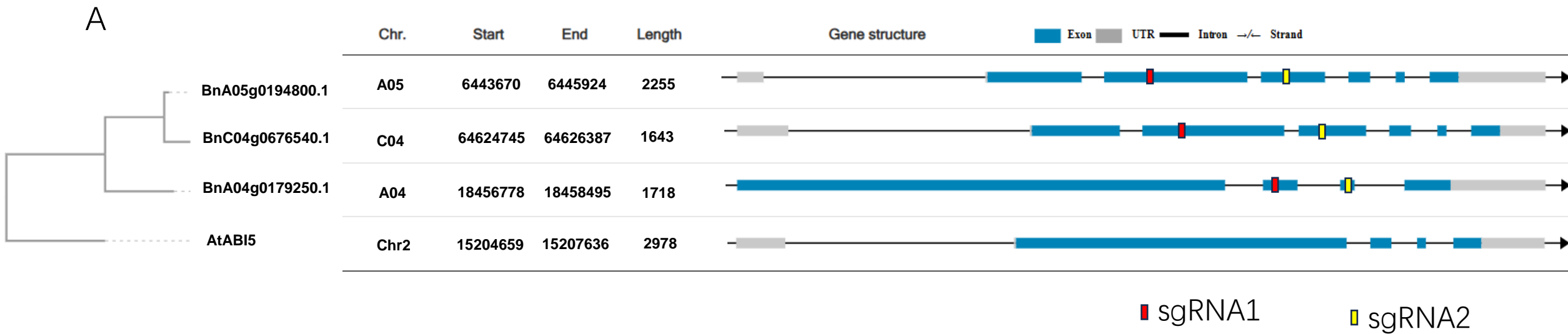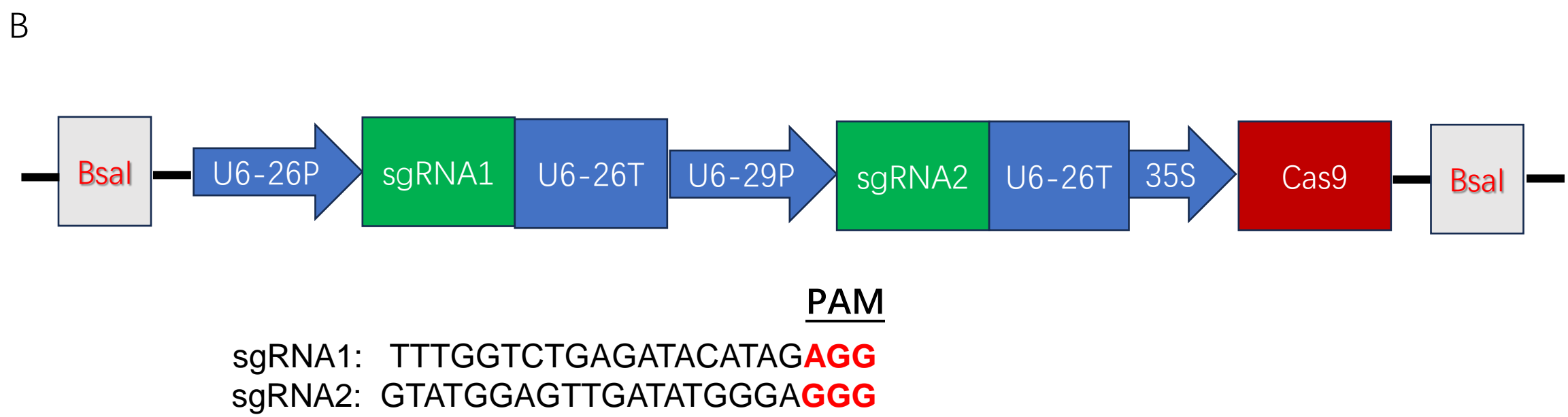

Supplement: Supplementary file 1 [file plants-14-01276-s001.zip › Supplementary Figure S1.pdf]

# GO enrichment of Upregulated DEGs

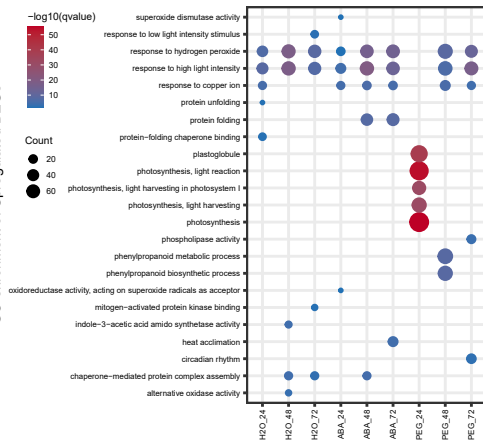

# KEGG enrichment of Upregulated DEGs

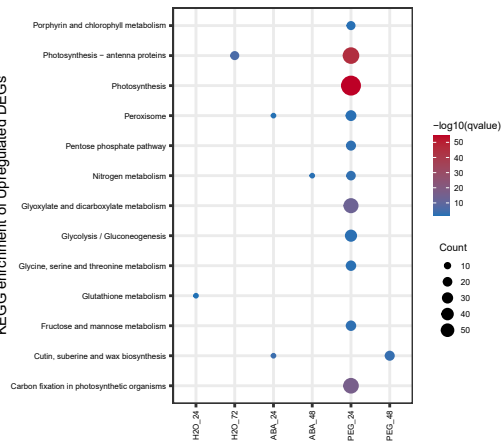

Supplement: Supplementary file 1 [file plants-14-01276-s001.zip › Supplementary Figure S3.pdf]
